# Receptor tyrosine kinases as druggable targets in glioblastoma: Do signaling pathways matter?

**DOI:** 10.1093/noajnl/vdab133

**Published:** 2021-09-17

**Authors:** Anna Qin, Anna Musket, Phillip R Musich, John B Schweitzer, Qian Xie

**Affiliations:** 1 Department of Biomedical Science, Quillen College of Medicine, East Tennessee State University, Johnson City, Tennessee, USA; 2 Department of Pathology, Quillen College of Medicine, East Tennessee State University, Johnson City, Tennessee, USA

**Keywords:** combination therapeutics, glioblastoma, receptor tyrosine kinase, resistance mechanisms, targeted therapy

## Abstract

Glioblastoma (GBM) is the most malignant primary brain tumor without effective therapies. Since bevacizumab was FDA approved for targeting vascular endothelial growth factor receptor 2 (VEGFR2) in adult patients with recurrent GBM, targeted therapy against receptor tyrosine kinases (RTKs) has become a new avenue for GBM therapeutics. In addition to VEGFR, the epidermal growth factor receptor (EGFR), platelet-derived growth factor receptor (PDGFR), hepatocyte growth factor receptor (HGFR/MET), and fibroblast growth factor receptor (FGFR) are major RTK targets. However, results from clinical Phase II/III trials indicate that most RTK-targeting therapeutics including tyrosine kinase inhibitors (TKIs) and neutralizing antibodies lack clinical efficacy, either alone or in combination. The major challenge is to uncover the genetic RTK alterations driving GBM initiation and progression, as well as to elucidate the mechanisms toward therapeutic resistance. In this review, we will discuss the genetic alterations in these 5 commonly targeted RTKs, the clinical trial outcomes of the associated RTK-targeting therapeutics, and the potential mechanisms toward the resistance. We anticipate that future design of new clinical trials with combination strategies, based on the genetic alterations within an individual patient’s tumor and mechanisms contributing to therapeutic resistance after treatment, will achieve durable remissions and improve outcomes in GBM patients.

**Key Points**

- We reviewed molecular structures and signaling pathways of 5 commonly targeted RTKs in GBM.
- We summarized genetic alterations of commonly targeted RTKs and their oncogenic functions.
- We discussed the clinical trial outcomes of RTK-targeting therapeutics, the potential resistance mechanisms, and combination strategies.

Glioblastoma, formerly known as glioblastoma multiforme (GBM) and regarded as a malignant astrocytic tumor, grade IV, is a highly infiltrative (also termed diffuse) glioma with a very poor prognosis. Most GBM arise de novo, that is, with no evidence for a pre-existing lower-grade glioma, and with a short clinical history. These are called “primary” GBM. Perhaps 5% of GBM arise from a previously apparent lower-grade, usually astrocytic, glioma. These tumors usually have a longer clinical history. If biopsied early in their course, they would merit a lower designation than grade IV. The most recent revision of the WHO monograph on the classification of brain tumors indicates that if any tumor with the morphologic attributes of GBM has an isocitrate dehydrogenase (IDH) mutation, they are classified as secondary GBM regardless of prior clinical course.^1^

Therapy of GBM is frustrated by numerous features that can be summarized generally as a failure of local control of the tumor. Unlike many other malignant tumors, distant metastases are very rare. Metastases via the cerebrospinal fluid (CSF) path, so called “drop metastases,” can occur but also are rare. The main obstacle to the treatments is the ability of GBM tumor cells to widely infiltrate adjacent and distant brain tissue and proliferate.^2^ For practical reasons wide excisions in the brain cannot be performed, so surgery usually is not curative. Optimal surgery, therefore, is considered to be the excision of 95% of the tumor defined radiologically followed by radiation therapy. Historically, this has resulted in an overall median survival of 14–16 months. The addition of temozolomide (TMZ) has resulted in a statistically significant increase in median overall survival among those with methylgaunine methyl transferase (MGMT) promoter hypermethylation (~45% of cases) out to ~21 months.^3^ Beyond its ability to infiltrate brain, GBM also is one of the most highly vascularized human cancers, which led to the development of VEGF inhibitors as an anti-angiogenesis therapy.

When the NCI and the National Human Genome Research Institute started the cancer genome atlas (TCGA) program, GBM became the first cancer type systemically analyzed using genomic approaches, demonstrating RTK signaling pathways as the most-altered core signaling pathways occurring in about 90% of GBM tumors.^4^ The most common genetic RTK alterations occur in the EGFR family. The 2 other RTKs frequently altered are the PDGFR and the MET tyrosine kinase pathways, further encouraging the development of RTK-targeting therapeutics in GBM. Multiple RTK inhibitors are being developed and evaluated in clinical trials; however, overall, they show lack of efficacy in treating GBM. In this review, we will introduce the key RTK targets in GBM, their genetic RTK alterations in gliomagenesis and progression (**Figure 1**, **Table 1**), the TKIs developed for treating GBM and their clinical efficacy in GBM patients (**Table 2**), as well as describing the challenges and opportunities of targeted therapy (**Figure 2**).

**Table 1.** Major Genetic RTK Alterations in GBM

RTK Alteration	Incidence	Clinical Relevance	Biological Functions
VEGFR2 amplification	VEGFR2 amplification detection varies from 6% to 17%.^5^	Increased VEGFR indicates angiogenesis and is associated to meschymal subtype of GBM and poor prognosis.^6^	VEGF promotes angiogenesis in GBM but also suppresses tumor cell invasion through a MET/VEGFR2 heterodimerization.^7^
EGFR amplification	About 45% of GBM have EGFR mutation or amplification.^4^	Indicates classical subtype of GBM and is associated to poor prognosis.^6,8^	Enhances neurosphere cell line growth in the presence of EGF/FGF.^6^
EGFRvIII	About 20% of GBM have EGFRvIII or other types of extracellular domain mutations.^4^	Controversal. Large-scale studies have not shown EGFRvIII as a prognostic marker for GBM.^9,10^	Upregulates DNA mismatch repair and increased sensitivity to TMZ.^11^
ERBB2 mutation	8% of GBM have ERBB2 mutation.^4^	High expression of ERBB2 associates to shorter survival time in GBM.^12^	EGFR depletion activates ERBB2 in GSCs, leading to resistance to EGFR inhibitors.^13^
PDGFRα amplification	13% of GBM show PDGFRα amplifications.^4^	PDGF signaling indicates the proneural subtype of GBM.^6,8^	Overexpression of PDGFRα mutant is associated to gliomagenesis.^14,15^
PDGFRβ overexpression	PDGFRβ, VEGFR2, PDGFRα, are overexpressed on the majority of endothelial cells in GBM.^16^		Overexpression initiates tumors in mice models, and contributes to glioma stem cell growth.^17^
MET amplification	About 4% GBM have MET amplification.^4^ However, 13-30% of GBM have MET overexpression.^18,19^	MET overexpression indicates poor prognosis in GBM.^20^	Overexpression of HGF/MET axis leads to glioma formation in mice.^21^
ZM fusion/ METex14	15% of secondary GBM have at least one ZM fusion protein.^22^ About 14% of secondary GBM have MET-exon-14 skipping.^23^	ZM fusion plus METex 14 associates to poor prognosis in secondary GBM.^23^	Exon 14 skipping removes the juxta-membrane domain of MET, generating cytosolic MET which is constitutively active in a ligand-independent manner but is sensitive to MET inhibitors.^23^
METΔ7-8	About 6% of high-grade gliomas, including 3.3% of GBM, have METΔ7-8 mutation.^24^	Presence indicates a high-grade glioma.^24^	Located predominantly in the cytosol, constitutively active and is sensitive to MET TKI.^24^
FGFR-TACC fusion	3% of GBM have an FGFR-TACC fusion protein, with FGFR3 and TACC3 as the most common fusion type (FGFR3-TACC3).^25,26^	FGFR3-TACC3 fusions in IDH wild-type glioma indicates sensitivity to FGFR inhibitors.^27^	FGFR3-TACC3 fusion protein transforms astrocytes into glioma cells in the mouse brain.^25^ FGFR-TACC changes metabolism of GBM cells.^26^

**Table 2.** RTK Inhibitors in Clinical Trials

Inhibitors	Targets	Study Title	Phase	Status
AZD4547	pan-FGFR	Treatment with AZD4547 for recurrent malignant glioma expressing FGFR-TACC gene fusion. NCT02824133	1，2	Completed
Afatinib	EGFR, EGFRvIII, ERBB2, ERBB4	Safety study of afatinib for brain cancer. NCT02423525	1	Active, not recruiting
Bevacizumab*	VEGF-A	Translational study of nivolumab in combination with bevacizumab for recurrent glioblastoma. NCT03890952	2	Recruiting
Cediranib	pan-VEGFR	Cediranib maleate and cilengitide in treating patients with progressive or recurrent glioblastoma. NCT00979862	1	Completed
		Cediranib maleate and olaparib compared to bevacizumab in treating patients with recurrent glioblastoma. NCT02974621	2	Active, not recruiting
		Temozolomide and radiation therapy with or without cediranib maleate in treating patients with newly diagnosed glioblastoma. NCT01062425	2	Active, not recruiting
Cetuximab	EGFR, EGFRvIII	Intraarterial infusion of erbitux and bevacizumab for relapsed/refractory intracranial glioma in patients under 22. NCT01884740	1, 2	Recruiting
		Super-selective Intra-arterial Repeated Infusion of Cetuximab for the Treatment of Newly Diagnosed Glioblastoma. NCT02861898	1, 2	Recruiting
Crizotinib	MET, ALK	Study to evaluate safety and activity of crizotinib with temozolomide and radiotherapy in newly diagnosed glioblastoma. NCT02270034	1	Active, not recruiting
		Study of the combination of crizotinib and dasatinib in pediatric research participants with diffuse pontine glioma and high-grade glioma. NCT01644773	1	Completed
Cabozantinib (XL184)	MET, VEGFR2	Study of multiple doses and regimens of XL184 (cabozantinib) in subjects with grade IV astrocytic tumors in first or second relapse. NCT01068782	2	Completed
		Pilot study of cabozantinib for recurrent or progressive high-grade glioma in children. NCT02885324	2	Recruiting
Dacomitinib	EGFR, ERBB2, HER4	Safety and efficacy of PF-299804 (dacomitinib), a pan-HER irreversible inhibitor, in patients with recurrent glioblastoma with EGFR amplification or presence of EGFRvIII mutation. A Phase II CT.^28^ NCT01520870	2	Completed
		PF-00299804 in adult patients with relapsed/recurrent glioblastoma. NCT01112527	2	Completed
Infigratinib	pan-FGFR	A phase 2 study of BGJ398 in patients with recurrent GBM.^29^ NCT01975701	2	Completed
		Infigratinib in recurrent high-grade glioma patients. NCT04424966	1	Recruiting
INCB28060	MET	INC280 combined with bevacizumab in patients with glioblastoma multiforme. NCT02386826	1	Active, not recruiting
Imatinib	PDGFR, ABL, KIT	Standard chemotherapy vs. chemotherapy guided by cancer stem cell test in recurrent glioblastom. NCT03632135	3	Recruiting
mAb806	EGFR, EGFRvIII	A study of ABT-806 in subjects with advanced solid tumor types.^30^ NCT01255657	1	Completed
Nimotuzumab	EGFR	Nimotuzumab plus radiotherapy with concomitant and adjuvant temozolomide for cerebral glioblastoma.^31^ NCT03388372	2	Completed
Osimertinib	EGFR	18F-FDG PET and osimertinib in evaluating glucose utilization in patients with EGFR activated recurrent glioblastoma. NCT03732352	2	Active, not recruiting
Onartuzumab	MET	A study of onartuzumab in combination with bevacizumab compared to bevacizumab alone or onartuzumab monotherapy in participants with recurrent glioblastoma.^32^ NCT01632228	2	Completed
PLB-1001	MET	Study of a c-Met inhibitor PLB1001 in patients with PTPRZ1-MET fusion gene positive recurrent high-grade gliomas.^23^ NCT02978261	1	Completed
Sunitinib	VEGFR1,2 PDGFRα, β	HGG-TCP (High grade glioma - tumor concentrations of protein kinase inhibitors). NCT02239952	Not Applicable	Recruiting
		A phase II/III study of high-dose, intermittent sunitinib in patients with recurrent glioblastoma multiforme. NCT03025893	2.3	Recruiting
		Combining sunitinib, temozolomide and radiation to treat patients diagnosed with glioblastoma. NCT02928575	2	Unknown

Most recent RTK inhibitor clinical trials in GBM were searched at www.Clinicaltrials.gov (2010 to present).

*At time of search, a total of 25 clinical trials are currently recruiting patients in United States.

**Figure 1.**
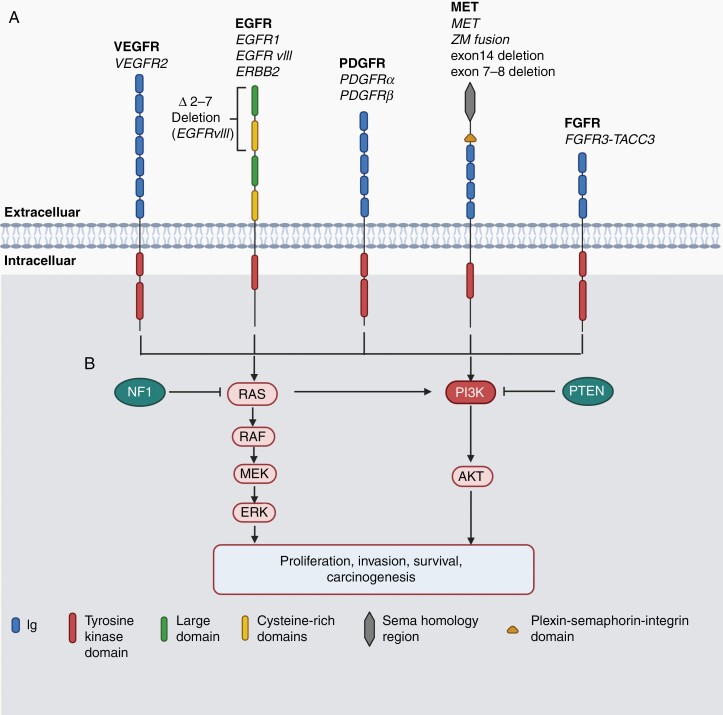
Critical RTK structure and signaling pathways in GBM. (A) RTK structure. The 5 RTK families most studied in GBM are shown. The variants with GBM genetic alterations are listed below the family names. All RTKs have a similar molecular architecture, which is characterized by an extracellular domain, a single transmembrane region and a cytoplasmic region consisting of a juxtamembrane domain, a tyrosine kinase (TK) domain and the carboxy terminal (modified from Lemmon, et al., with permission). (B) RTK signaling pathway. RTKs can be activated through ligand-dependent or ligand-independent mechanisms, leading to receptor dimerization and phosphorylation at the TK domains. RTK phosphorylation further triggers downstream signaling pathways that activate or repress genes involved in proliferation, invasion, survival and carcinogenesis. An elevation of the RTK-mediated RAS and PI3K signaling pathway (RTK/RAS/PI3K) is the most frequent signaling alteration, occurring in about 90% of GBM patient specimens. As in other cancer types, additional mutation, or deletion of tumor suppressor genes such as NF1 and PTEN further accelerates RTK/RAS/PI3K activity, promoting glioma initiation and malignant progression.

**Figure 2.**
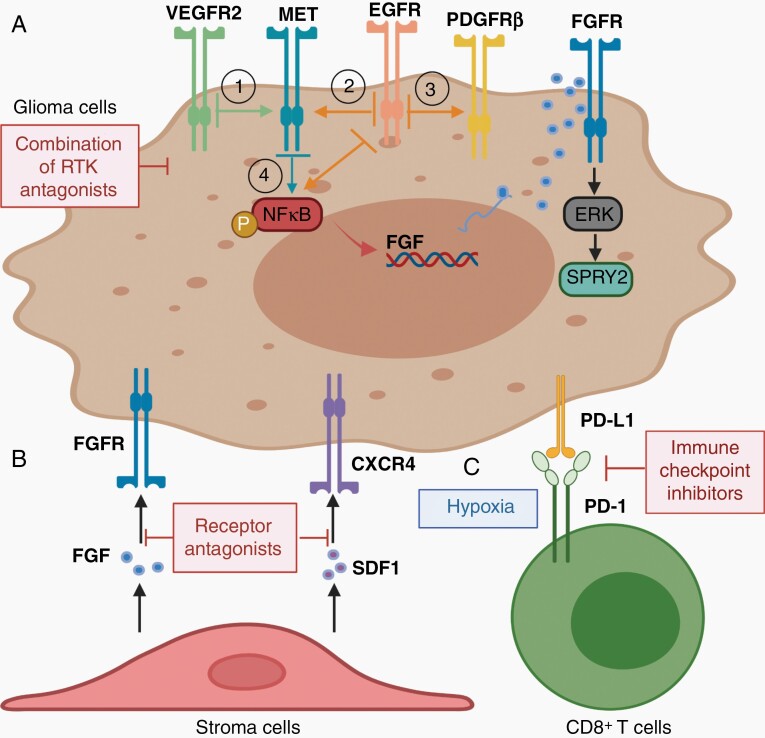
Mechanisms of resistance and potential combination strategy. Acquired resistance to RTK-targeting reagents may involve 3 mechanisms: (A) through compensatory upregulation of other RTKs. It is common that inhibiting a single RTK signaling may cross activate another pathway to sustain tumor proliferation and invasion; thus, a combination of multiple TKIs will improve the therapeutic efficacy (see the text for more details); (B) through stroma-mediated soluble factor secretion. Tumor cells under treatment may select to respond to the signals from the microenvironment that are favored for survival, leading to tumor recurrence. Thus, TKIs in combination with those receptor antagonists may improve the therapeutic efficacy; (C) through hypoxia-mediated immune checkpoint activation. After anti-angiogenic therapy, the consequent hypoxic environment may upregulate immune checkpoint signaling activation in the tumor, leading to T cell exhaustion. Thus, TKIs in combination with checkpoint inhibitors may improve the therapeutic efficacy.

## RTK Structure and Signaling Pathways

RTKs are a subclass of tyrosine kinases that lead to phosphorylation at the intracellular tyrosine residues of a transmembrane receptor protein using adenosine triphosphate (ATP). The human RTK family has 58 known members; these are further classified into 20 multi-member subfamilies including EGFR, VEGFR, PDGFR, FGFR, and MET (**Figure 1**), which are the most commonly studied RTKs in glioma initiation and progression.^4^

Structurally, all RTKs are integral membrane proteins composed of an extracellular domain, which contains the ligand-binding site, a single transmembrane domain, and an intracellular domain, which includes tyrosine kinase (TK) activation sites (**Figure 1A**).^33^ RTK activation is triggered by binding of the ligand, which leads to receptor dimerization and auto-phosphorylation at the TK sites. These phosphorylated residues further create docking sites for recruiting adaptor proteins, leading to activation of downstream signaling. Activated RTKs recruit small G-proteins (RAS) to the cell membrane, leading to RAS activation of multiple downstream signaling pathways, including the RAF/MEK/ERK and PI3K/AKT signaling pathways which control cellular proliferation, invasion, survival, and carcinogenesis (**Figure 2B**). Genetic alteration of RTKs is common in GBM. These alterations can lead to constitutive RTK activation through ligand-dependent or ligand-independent mechanisms, resulting in RTK/PI3K/AKT signaling being the most elevated core pathway in tumors.^4^ Additional mutation or deletion of tumor suppressor genes such as TP53, NF1 and PTEN further accelerates RTK/RAS/PI3K activity, promoting glioma initiation, and malignant progression (**Figure 1**).^4,34^

## Genetic RTK Alteration and Gliomagenesis

Beginning in 2006, TCGA launched a cancer genomics program to systematically characterize the molecular classification of cancers for personalized medicine. With next generation sequencing as an approach, these studies identified a comprehensive landscape of genetic alterations in line with core signaling pathways; this led to the classification of GBM into 4 molecular subtypes, classical, mesenchymal, proneural, and neural, each associated to clinical prognosis.^4,6,8^ From the genetics perspective, EGFR mutation and amplification are the most frequent RTK alterations, occuring in about 45% of GBM tumors. EGFRvIII, a mutant of EGFR with an in-frame deletion of exons 2–7 from the extracellular region, along with other extracellular domain mutations, occurs in about 20% of GBM samples, and ~70% of these also show EGFR amplification. The ERBB2 mutant lacking the ligand binding domain occurs in ~8% of GBM patients.^4^ PDGFRα amplification also is common and often occurs with EGFR amplification.^4,35^ Both PDGFRα, PDGFRβ and VEGFR2 are overexpressed on the majority of endothelial cells in GBM.^16^ While MET overexpression occurs in 20%–30% high-grade glioma,^18,20,36^ about 4% primary GBM is found to have MET amplification.^4^ About 3% of GBM have a constitutively-active form of MET mutation with exon 7 and 8 deletion (METΔ7-8) resulting in a MET protein that is predominantly cytosolic and ligand independent.^24^ In addition, exon skipping during MET pre-mRNA splicing can result in a METex14 deletion, which removes the intracellular juxtamembrane domain of MET protein; this domain is necessary for MET protein degradation.^23^ METex14 frequently occurs with a PTPRZ1-MET (ZM) gene fusion, which is present in approximately 15% of secondary GBM and is associated with poor prognosis.^23^ The most common FGFR mutation is a gene rearrangement between FGFR3 and transforming acidic coiled-coil-containing gene 3 (TACC3), yielding the FGFR3-TACC3 fusion protein in ~ 3% GBM.^37^ Commonly, RTK amplifications and mutations result in overexpression of the wild-type or mutant receptor proteins, which often indicates a poor prognosis in GBM patients (see Figure 1, Table 1 for summary). More importantly, amplifications of multiple RTKs within the same tumor is frequent in GBM,^34,35^ demonstrating a heterogenous disease and the need for tailored targeting of RTK signaling pathways.

During normal neurogenesis, neural stem cells (NSCs) are primarily located in the hippocampus and subventricular zone (SVZ) where they proliferate into multi-lineage progenitor cells and differentiate into neurons, astrocytes, and oligodendrocytes to populate the nervous system.^21^ However, glioma stem cells (GSCs) may arise from normal NSCs or neural progenitor cells that harbor genetic alterations, such as amplifications and mutations, resulting in oncogene overexpression and leading to gliomagenesis. Early studies of EGFR signaling showed that neural progenitors from the adult SVZ respond to EGFR activation with enhanced proliferation and migration.^38,39^ Using the sleeping beauty transposon system Wiesner *et al*. showed that injecting a mixture of plasmids coding for EGFR*vIII*, NRAS and AKT into the lateral ventricle of neonatal mouse brain induced glioma cells arising from the SVZ, forming tumors with pathology resembling human GBM.^40^ With MET RTK, Qin et al. demonstrated that transgenic mice overexpressing the human HGF/MET axis along with p53 inhibition induced GBM in mice.^21^ Moreover, in mice deficient in the tumor suppressor genes Ink4b and Arf irradiation-induced DNA damage triggered glioma formation with Met amplification as the most significant oncogenic event.^41^ In all these 3 animal models, glioma arising from EGFR*vIII* or MET activation expressed GSC markers, supporting the concept that transforming NSCs into GSCs is critical to GBM development. While PDGFRα overexpression is associated with poor prognosis, a human GBM-derived PDGFRα mutant with deletion of exons 8 and 9 was found to transform normal Rat1 cells into oncogenic cells which induced tumor formation in nude mice.^14^ A recent study using recombinant lentiviruses expressing both PDGFβ and a short hairpin RNA targeting Cdkn2a induced proneural GBM following intracranial injection into the adult immunocompetent mice.^17^ Although genetic FGFR alteration is rare, gene constructs derived from GBM patients expressing the FGFR3-TACC3 fusion demonstrated oncogenic activity in transforming astrocytes into glioma cells in the mouse brain.^25,27^ All these results suggest that RTKs not only play important roles in brain development, but also can be driving forces for glioma formation, providing a strong rationale for developing therapeutics targeting of RTKs.

## Targeted Therapeutics Against RTKs in GBM

### VEGFR

GBM is one of the most aggressive tumors with structurally and functionally abnormal vasculature. In 1996, Chen et al. introduced an antisense construct against VEGF expression into glioblastoma cells and showed that inhibiting VEGF expression reduced vasculature formation and suppressed tumor growth, suggesting that VEGF signaling plays a major angiogenic role in glioblastoma.^42^ In 2005, bevacizumab, a humanized monoclonal anti-angiogenic antibody which binds to VEGF-A, prevents its interaction with VEGFR1 and VEGFR2 on the surface of endothelial cells, was first tested in clinical trials in patients with recurrent GBM.^43^ In 2009, bevacizumab became the first FDA-approved targeting therapy for treating recurrent GBM. However, although bevacizumab showed improved progression-free survival (PFS) it had no overall survival (OS) benefit, indicating that targeting VEGFR alone is not sufficient for inhibiting tumor growth.^44^

Beside bevacizumab, small molecule inhibitors against VEGFR were developed for treating GBM with cediranib and sunitinib showing the most promising results. Cediranib is a potent ATP-competitive inhibitor against VEGFR2 with additional activity against PDGFRβ and c-Kit.^45^ Experimentally, cediranib not only prevented new vessel formation, but also induced vascular regression in xenograft tumor models. However, similar to bevacizumab, a phase II clinical trial of cediranib monotherapy with recurrent GBM showed encouraging radiographic response, 6-month PFS (PFS-6) time with manageable toxicity, but no OS benefit (NCT00305656).^46^ Sunitinib is another oral multi-target TKI designed for anti-angiogenesis, mainly targeting VEGFR and, even more effectively, PDGFRα and β.^47^ Although preclinical data showed sunitinib inhibited angiogenesis and prolonged survival in mice bearing orthotopic GBM, the effect seemed less potent than bevacizumab treatment,^48^ a clinical phase II trial of recurrent GBM showed that daily sunitinib did not prolong PFS in bevacizumab-naive nor resistant patients (NCT00923117).^49^

The lack of clinical efficacy of anti-VEGF agents raised the importance of studying mechanisms of tumor resistance and recurrence and developing combination strategies to improve the clinical efficacy. Inhibition of the VEGF pathway was shown to increase tumor cell invasion through MET pathway activation in animal models, while heterodimer formation between MET and VEGFR (specifically VEGFR2) was found to negate both invasiveness and angiogenesis activity.^7^ Based on these results, a recent clinical trial has shown that a combination of bevacizumab and the anti-MET antibody onartuzumab significantly improved both PFS and OS in the mesenchymal subtype of recurrent GBM patients with high HGF expression (NCT01632228).^32^ Previous studies also suggest that anti-VEGF agents may induce PD-1 (programmed cell death protein 1) expression on CD8+T cells and PD-L1 (PD-1 ligand) in glioma cells, thus contributing to an immunosuppressive microenvironment.^50^ In this case, application of bevacizumab may revert the T cell-mediated immune response against tumor cells as well as improve the efficacy of immune checkpoint inhibitors. Currently, there is a phase II translational study of nivolumab, a monoclonal antibody that blocks immune checkpoint PD-1, in combination with bevacizumab for recurrent glioblastoma (NCT03890952). Despite the development of various new drugs against GBM, bevacizumab remains the most utilized drug evaluated in clinical trials against GBM. At the time of this literature search, 25 clinical trials of bevacizumab in combination with other agents are currently recruiting patients in the United States (**Table 2**).

### EGFR and ERBB2

Given the high prevalence of EGFR and ERBB2 amplification/overexpression in GBM patients and the unique EGFRvIII mutation that occurs in tumors rather than normal brain, EGFR and its mutants have been attractive targets for developing GBM treatments (**Table 1**).

At present, there are 3 generations of EGFR inhibitors. The first-generation inhibitors (gefitinib, lapatinib, and erlotinib) were designed as reversible competitors for the ATP-binding site in the EGFR kinase domain. However, results from phase II clinical trials demonstrated only marginal therapeutic response in primary or recurrent GBM patients, either as monotherapy or in combination therapy.^51–53^ To improve efficacy, the second-generation EGFR inhibitors (dacomitinib and afatinib) were designed to bind to the receptor irreversibly.^54^ Dacomitinib is an orally taken EGFR inhibitor targeting EGFR, ERBB2 and ERBB4, and demonstrated activities against mutant EGFR and gefinitib-resistant lung cancer cells in vitro and in animal models.^55^ This suggests a higher activity than earlier EGFR inhibitors in treating GBM with EGFR amplification. Dacomitinib did impair the growth of tumors containing EGFR amplification in GBM xenograft models,^56^ but only showed limited activity in clinical trials with recurrent GBM containing EGFR amplification (NCT01520870).^28^ This study found no significant improvement of PFS-6, regardless of EGFRvIII status. Furthermore, the toxicity of dacomitinib raised safety concerns. Afatinib is another potent, orally taken ATP-competitive inhibitor targeting EGFR, EGFRvIII, ERBB3 and ERBB4.^57,58^ Although it showed inhibitory efficacy against GBM cells harboring EGFRvIII and EGFR R108K mutations,^57,59^ results from completed phase I/II clinical trials showed that afatinib alone had limited activity with recurrent GBM patients, and the addition of temozolomide did not improve the PFS-6 rate or the median PFS (NCT00727506).^60^ Thus, the overall antitumor activities of second-generation EGFR inhibitors were comparable to those of the first generation. The third-generation irreversible inhibitor osimertinib is designed to target EGFR T790M, a common mutation in non-small-cell lung cancer (NSCLC), and reduced the resistance that developed after treatment with first- or second-generation inhibitors.^61^ Preclinically, osimertinib inhibited EGFRvIII-mediated downstream signaling in GSCs, and significantly increased the survival time in mice bearing intracranial EGFRvIII-positive tumors.^62^ Osimertinib efficiently crosses the blood–brain barrier (BBB), making it an attractive candidate for inhibiting EGFR in GBM. Osimertinib is now in a phase I/II clinical trial in GBM (NCT03732352) (**Table 2**).

Cetuximab and nimotuzumab are monoclonal antibodies developed for targeting EGFR. Cetuximab binds to EGFR with high affinity, competes for ligand binding, and down-regulates cell-surface receptor expression.^63,64^ Nimotuzumab binds to EGFR without intrinsic stimulating activity and with lower affinity than cetuximab, thus binding more specifically to EGFR-overexpressing cells.^65^ Cetuximab showed inhibitory activity for inhibiting EGFR-amplified GBM cells in vitro and in vivo^66–68^ but not in an early phase II clinical trial with recurrent GBM patients (NCT00463073). When in combination with bevacizumab and irinotecan, cetuximab showed well-tolerated toxicity, but the overall response is not superior to single-agent bevacizumab or the combination of bevacizumab plus irinotecan.^69^ In contrast, a phase II clinical trial showed that nimotuzumab in combination with standard radiotherapy (RT) and TMZ was well-tolerated and prolonged median OS and PFS in patients with newly-diagnosed, EGFR-positive GBM (NCT03388372).^31^ Because EGFRvIII is a mutant of EGFR lacking the extracellular ligand-binding domain, mAb806, a specific antitumor antibody against EGFRvIII, was generated to its epitope of EGFR residues 287-302.^70^ A phase I clinical trial showed that mAB806 was safe in GBM patients (NCT01255657).^30^ In addition, the mouse-human chimeric monoclonal antibody 806 (ch806), which was constructed by linking the heavy and light chain variable regions of murine mAb806 to human gamma-1 and kappa constant regions, respectively, was developed.^71^ Ch806 was evaluated in a phase I clinical trial with cancer patients harboring EGFRvIII, showing good safety and specificity of targeting EGFRvIII but not wild-type EGFR.^72^

### PDGFRα

Among the RTKs, EGFR and PDGFRα were the 2 earliest ones identified to have amplifications in gliomas.^73^ Early study also demonstrated a human GBM-derived PDGFRα mutant transcript with an in-frame deletion of exons 8 and 9 in the extracellular domain (PDGFRα (Δ8,9)) that transformed Rat1 cells into oncogenic cells capable of inducing tumor formation in nude mice.^14^ Further study with GBM also showed that a PDGFRα with a transmembrane domain mutation V536E stimulated Ba/F3 cell growth and signaling via ERK, which can be strongly inhibited by blocking PDGFR activation.^15^

Imatinib is a TKI mainly targeting PDGFR, KIT and ABL.^74^ Despite its superior efficacy for treating chronic myloid leukemia,^75^ imatinib only showed limited anti-tumor activity in GBM clinical trials, either as a monotherapy^76^ or in combination with hydroxyurea, a ribonucleotide reductase inhibitor.^77^ By analyzing TCGA datasets, Song et al.^78^ found that the expression of 3 RTKs, that is, ERBB3, insulin growth factor 1 receptor (IGF1R), and transforming growth factor-β receptor 2 (TGF-βR2), were positively correlated with that of PDGFR in GBM samples, suggesting a signaling cross talk among these RTKs. Experimentally, combination of a PDGFR TKI with another inhibitor targeting either ERBB3 or IGF1R more potently suppressed the growth of GBM cells than either inhibitor alone. Therefore, identifying the RTKs responsible for resistance to PDGFR inhibitors may synergistically enhance the anti-glioma efficacy.^78^ SHP-2 is a non-receptor protein tyrosine phosphatase encoded by the *PTPN11* gene that is critical for PDGFRα-driven gliomagenesis. Recently, Sang et al. showed that specifically targeting SHP-2 potently inhibited GBM cells with PDGFRα activation, providing a new candidate for therapeutically targeting the PDGFR signaling pathway.^79^

### MET

MET has been a promising druggable target in many cancer types for decades and is well-known for promoting cancer proliferation and invasion.^80^ Recent studies not only show that multiple MET genetic alterations are found in GBM and are associated to poor prognosis, but also that activation of the HGF/MET axis contributes to gliomagenesis^21,41^ (**Table 1**). As described above, inhibiting the angiogenesis pathway through VEGF sequestration results in MET activation, converting glioma into a more invasive phenotype.^7^ All these results provide a strong rationale for targeting MET in GBM.

MET small molecule inhibitors that have been evaluated in clinical trials for treatment of GBM include crizotinib, PLB-1001, INCB28060, and cabozantinib (**Table 2**). Crizotinib is a dual-targeted TKI for MET and ALK, and is FDA approved for the treatment of ALK-rearranged NSCLC.^81,82^ Based on biochemical and cellular data of over 120 kinases, crizotinib selectively targeted MET and ALK at pharmacologically relevant concentrations more potently than other kinases.^81^ Preclinically, crizotinib specifically inhibited MET-positive GSCs derived from GBM patients in vitro, and tumor growth in vivo.^83^ To date, there are 2 phase I clinical trials to evaluate the safety and activity of crizotinib with temozolomide and radiotherapy for newly diagnosed GBM (NCT02270034), and to evaluate the tolerable dose of crizotinib and dasatinib in pediatric patients with diffuse pontine glioma and high-grade glioma (NCT01644773). Compared with crizotinib, PLB-1001 binds to the kinase domain of MET plus 2 additional interaction sites, leading to a higher binding affinity and a better inhibitory effect. Functionally, PLB-1001 not only demonstrates a good BBB permeability, but also has a higher efficacy than crizotinib in inhibiting MET-driven GBM tumor growth in mice.^23^ Importantly, a phase I clinical trial has shown that PLB-1001 monotherapy achieved safety and a partial response for secondary GBM or grade III glioma patients with ZM fusion and/or METex14. These findings indicate that PLB-1001 is a highly selective, efficient, and BBB-permeable MET kinase inhibitor for treating MET-driven GBM patients (NCT02978261). INCB28060 is a small molecule inhibitor with picomolar enzymatic potency and is highly specific for MET with more than 10,000-fold selectivity over a large panel of other human kinases.^84^ Functionally, INCB28060 showed potent inhibition of MET-mediated signaling cascades in various cancer cells, and preferentially inhibited tumors with MET amplification.^84,85^ Currently, a phase I clinical trial is evaluating the safety and efficacy of the combination of INC28060 and bevacizumab in GBM patients with previous treatment and those with unresectable GBM (NCT02386826). Cabozantinib (XL184) is a multitarget TKI with potent activity against MET and VEGFR2 and is FDA approved for treating hepatocellular carcinoma patients previously treated with sorafenib, a VEGF inhibitor. However, based on the results from a completed Phase II clinical trial with progressive GBM patients, cabozantinib monotherapy showed evidence of clinical activity, but failed to meet the predefined statistical target for success (NCT00704288).^86^ Current studies have focused on multiple doses and regiments of cabozantinib in recurrent GBM and high-grade glioma in children (**Table 2**).

### FGFR

FGFR amplification in GBM is rare; approximately 3% of GBM harbor an oncogenic chromosomal translocation of an FGFR1 or FGFR3 gene to the coding domain of TACC1 or TACC3.^25,27^ In the case of a FGFR3-TACC3 fusion, the intracellular FGFR tyrosine domain is fused to the TACC coiled-coil domain, resulting in constitutive FGFR kinase activation. While early studies have demonstrated that FGFR3-TACC3 fusion protein may transform astrocytes into glioma cells in the mouse brain, a recent study showed that tumors harboring FGFR3-TACC3 fusion rely on mitochondrial oxidative phosphorylation for metabolism.^26^ Furthermore, Day et al. showed that GBM cells can evade EGFR and MET inhibition via FGFR-SPRY2 bypass signaling, and that adding a FGFR inhibitor may increase GBM response to EGFR and MET inhibition.^87^ Both studies have highlighted the therapeutic potential for treating tumors with FGFR3-TACC3 fusion.

Both infigratinib^88^ and AZD4547^89^ are highly specific pan-FGFR kinase inhibitors with a higher potency against FGFR1-3 than FGFR4. These 2 inhibitors show significant efficacy for inhibiting FGFR alteration-mediated cancer cell proliferation in vitro and tumor growth in vivo. A recent phase II clinical trial with recurrent GBM patients harboring FGFR alterations shows that infigratinib induced a partial response or stable disease in approximately one-third of 26 patients, calling for additional clinical studies to include biomarker and combination strategies (NCT01975701).^29^ There is also a phase I/II study evaluating AZD4547 efficacy in recurrent GBM expressing FGFR-TACC gene fusion (NCT02824133).

Erdafitinib (JNJ-42756493) is a potent, orally active small molecule with potent tyrosine kinase inhibitory activity against all 4 FGFR family members.^90^ A recent study with IDH wild-type glioma showed that erdafitinib potently inhibited the proliferation of glioma cells harboring FGFR3-TACC3 fusions in vitro, and tumor growth in vivo. More importantly, 2 patients with FGFR3-TACC3 rearrangements who received erdafitinib demonstrated clinical improvement with stable disease and minor response, validating FGFR3-TACC3 as a therapeutic target for treatment with FGFR inhibitors.^27^

## Challenge and Opportunity

Since bevacizumab’s FDA approval for treating GBM, targeted therapy has opened a new avenue in neuro-oncology; however, resistance due to limited clinical efficacy is the biggest challenge, leading to tumor recurrence often with a more malignant phenotype. Thus, a thorough understanding of the mechanisms leading to resistance after the therapeutic treatment is critical and shall provide strategies for targeted therapy to improve the clinical efficacy.

RTK-mediated signaling pathways share multiple downstream elements (**Figure 1B**). Inhibiting one RTK often leads to the compensatory upregulation the other RTKs, resulting in signaling bypass and tumor resistance (**Figure 2A**). Among the RTKs, the HGF/MET axis frequently has been reported to be responsible for resistance to inhibition of other RTKs in various types of cancer and in GBM. Lu et al. reported that VEGF promotes angiogenesis in GBM but also suppresses tumor cell invasion through a MET/VEGFR2 heterodimerization. Blocking VEGF signaling may restore MET pathway activity as a compensatory signaling (**Figure 2A-1**), leading to tumor recurrence with a more invasive phenotype. Consequently, inhibition of MET in GBM mouse models treated by bevacizumab resulted in substantial survival benefit.^7^ Using a preclinical GBM mouse model, Jun et al. showed that gefinitib inhibition of EGFR resulted in MET overexpression as the most significant transcriptional change (**Figure 2A-2**). Furthermore, adding a MET inhibitor overcame the resistance to gefinitib.^91^ In addition, Akhavan et al. reported that EGFR inhibition promotes PDGFRβ upregulation in glioma cells (**Figure 2A-3**) and that the combination of EGFR and PDGFRβ inhibitors resulted in more potent antitumor activity in preclinical glioma animal models than either treatment alone.^92^ As mentioned above, GBM cells treated with EGFR and MET inhibitors may activate NF-κB signaling pathway, resulting in autocrine FGFR activation and SPRY2 overexpression for cellular resistance (**Figure 2A-4**). Thus, an FGFR inhibitor increased GBM response to combined EGFR and MET inhibition in preclinical GBM models in vivo.^87^

Tumor cells also may escape from drug sensitivity by exposure to growth factors usually secreted by the neighboring stromal cells, which is well accepted as environment-mediated drug resistance (**Figure 2B**).^93^ Early clinical trials have observed that tumor vasculature in GBM patients may be normalized by VEGF inhibitors; however, this was reversible after a 1-month treatment. When resistance occurs, basic plasma levels of FGF and SDF1α were increased as were circulating endothelial cells, suggesting a microenvironmental role in promoting resistance and tumor recurrence.^16^ Although GBM is considered as immunologically “cold” tumors which do not respond to immune checkpoint inhibitors well, studies have suggested that anti-angiogenic therapy may change the tumor microenvironment into immunological favorable “hot” tumors. In particular, the hypoxia environment resulted from bevacizumab treatment may upregulate PD-L1 expression in tumor cells and PD-1 expression in CD8+T cells, leading to effector T cell exhaustion and tumor regrowth (**Figure 2C**).^50^ This has led to the combination strategy of using immune checkpoint inhibitors to improve the bevacizumab efficacy for treating GBM patients (NCT03890952).

Another major challenge is that preclinical efficacy data does not predict clinical trial results well. Of note, preclinically, most RTK-targeting reagents are evaluated using cell lines and animal models harboring unique genetic modifications with selective pathway activations. These models are well accepted for testing the specificity and therapeutic efficacy of inhibitors against various RTK targets, but do not truly propagate the nature of tumor heterogeneity clinically. Heterogeneity among GBM has long been recognized both pathologically and molecularly. In particular, the WHO Classification of Central Nervous System Tumors is updated and re-issued this year (2021) with molecular pathways distinguished in what used to be lumped together as “GBM.” ^94^ An integrated diagnosis with additional molecular information will be the first step toward patient selection that informs therapeutic target selection. For example, a conventional histological GBM that is IDH mutated with CDKN2A/B homozygous deletion will not be called a “GBM” but an “Astrocytoma, IDH mutant, grade 4.” Note the (intentional) Arabic numeral. Similarly, a diffusely invading IDH wild-type astrocytic neoplasm without necrosis or microvascular proliferation but showing either EGFR amplification, TERT promoter mutation or a combined loss of chromosome 10 plus gain of chromosome 7 will be called a “GBM” despite the lack of histological hallmarks. Pediatric type diffusely infiltrating gliomas will be recognized regardless of patient’s age, marked by a host of markers that are possible targets (low grade: FGFr1 mutants, BRAF pV600E mutant; high grade: H3K27 altered, H3-G24 mutant), and H3 wild-type plus IDH wild-type diffuse pediatric gliomas that have multiple characterized molecular pathways often involving amplifications (PDGFRA, EGFR, MYCN). GBM harboring multiple genetic modifications within the same tumor is common. Thus, developing strategies for patient selection will be the key to the future success of RTK-targeting therapeutics.

Finally, the reduced capacity of some of these small molecule inhibitors to cross the BBB is also a critical challenge to the clinical efficacy. Thus, clinical development of novel drug delivery approaches to bypass the BBB is essential to the success of targeted therapeutics.^95^ We anticipate that development of personalized treatment protocols based on the individual patient’s genetic alterations, a better understanding of resistance mechanisms, along with enhanced drug delivery approaches will provide the best opportunity for achieving durable remissions and improved outcomes in GBM patients.

## References

[1] Louis DN , Perry A, Reifenberger G, et al. The 2016 World Health Organization classification of tumors of the central nervous system: a summary. Acta Neuropathol. 2016;131(6):803–820. 27157931 10.1007/s00401-016-1545-1

[2] Burger PC , Kleihues P. Cytologic composition of the untreated glioblastoma with implications for evaluation of needle biopsies. Cancer. 1989;63(10):2014–2023. 2539242 10.1002/1097-0142(19890515)63:10<2014::aid-cncr2820631025>3.0.co;2-l

[3] Hegi ME , Diserens AC, Gorlia T, et al. MGMT gene silencing and benefit from temozolomide in glioblastoma. N Engl J Med. 2005;352(10):997–1003. 15758010 10.1056/NEJMoa043331

[4] Cancer Genome Atlas Research N. Comprehensive genomic characterization defines human glioblastoma genes and core pathways. Nature. 2008;455(7216):1061–1068. 18772890 10.1038/nature07385 PMC2671642

[5] Puputti M , Tynninen O, Sihto H, et al. Amplification of KIT, PDGFRA, VEGFR2, and EGFR in gliomas. Mol Cancer Res. 2006;4(12):927–934. 17189383 10.1158/1541-7786.MCR-06-0085

[6] Phillips HS , Kharbanda S, Chen R, et al. Molecular subclasses of high-grade glioma predict prognosis, delineate a pattern of disease progression, and resemble stages in neurogenesis. Cancer Cell. 2006;9(3):157–173. 16530701 10.1016/j.ccr.2006.02.019

[7] Lu KV , Chang JP, Parachoniak CA, et al. VEGF inhibits tumor cell invasion and mesenchymal transition through a MET/VEGFR2 complex. Cancer Cell. 2012;22(1):21–35. 22789536 10.1016/j.ccr.2012.05.037 PMC4068350

[8] Verhaak RG , Hoadley KA, Purdom E, et al. Integrated genomic analysis identifies clinically relevant subtypes of glioblastoma characterized by abnormalities in PDGFRA, IDH1, EGFR, and NF1. Cancer Cell. 2010;17(1):98–110. 20129251 10.1016/j.ccr.2009.12.020 PMC2818769

[9] Weller M , Kaulich K, Hentschel B, et al.; German Glioma Network. Assessment and prognostic significance of the epidermal growth factor receptor vIII mutation in glioblastoma patients treated with concurrent and adjuvant temozolomide radiochemotherapy. Int J Cancer. 2014;134(10):2437–2447. 24614983 10.1002/ijc.28576

[10] Felsberg J , Hentschel B, Kaulich K, et al. Epidermal growth factor receptor variant III (EGFRvIII) positivity in EGFR-Amplified Glioblastomas: prognostic role and comparison between primary and recurrent tumors. Clin Cancer Res. 2017;23(22):6846–6855. 28855349 10.1158/1078-0432.CCR-17-0890

[11] Struve N , Binder ZA, Stead LF, et al. EGFRvIII upregulates DNA mismatch repair resulting in increased temozolomide sensitivity of MGMT promoter methylated glioblastoma. Oncogene. 2020;39(15):3041–3055. 32066879 10.1038/s41388-020-1208-5 PMC7142016

[12] von Achenbach C , Weller M, Szabo E. Epidermal growth factor receptor and ligand family expression and activity in glioblastoma. J Neurochem. 2018;147(1):99–109. 29953622 10.1111/jnc.14538

[13] Clark PA , Iida M, Treisman DM, et al. Activation of multiple ERBB family receptors mediates glioblastoma cancer stem-like cell resistance to EGFR-targeted inhibition. Neoplasia. 2012;14(5):420–428. 22745588 10.1596/neo.12432 PMC3384429

[14] Clarke ID , Dirks PB. A human brain tumor-derived PDGFR-alpha deletion mutant is transforming. Oncogene. 2003;22(5):722–733. 12569364 10.1038/sj.onc.1206160

[15] Velghe AI , Van Cauwenberghe S, Polyansky AA, et al. PDGFRA alterations in cancer: characterization of a gain-of-function V536E transmembrane mutant as well as loss-of-function and passenger mutations. Oncogene. 2014;33(20):2568–2576. 23752188 10.1038/onc.2013.218

[16] Batchelor TT , Sorensen AG, di Tomaso E, et al. AZD2171, a pan-VEGF receptor tyrosine kinase inhibitor, normalizes tumor vasculature and alleviates edema in glioblastoma patients. Cancer Cell. 2007;11(1):83–95. 17222792 10.1016/j.ccr.2006.11.021 PMC2748664

[17] Rahme GJ , Luikart BW, Cheng C, Israel MA. A recombinant lentiviral PDGF-driven mouse model of proneural glioblastoma. Neuro Oncol. 2018;20(3):332–342. 29016807 10.1093/neuonc/nox129 PMC5817944

[18] Xie Q , Bradley R, Kang L, et al. Hepatocyte growth factor (HGF) autocrine activation predicts sensitivity to MET inhibition in glioblastoma. Proc Natl Acad Sci USA. 2012;109(2):570–575. 22203985 10.1073/pnas.1119059109 PMC3258605

[19] Kwak Y , Kim SI, Park CK, Paek SH, Lee ST, Park SH. C-MET overexpression and amplification in gliomas. Int J Clin Exp Pathol. 2015;8(11):14932–14938. 26823824 PMC4713610

[20] Petterson SA , Dahlrot RH, Hermansen SK, et al. High levels of c-Met is associated with poor prognosis in glioblastoma. J Neurooncol. 2015;122(3):517–527. 25800004 10.1007/s11060-015-1723-3

[21] Qin Y , Musket A, Kou J, et al. Overexpression of HGF/MET axis along with p53 inhibition induces de novo glioma formation in mice. Neurooncol Adv. 2020;2(1):vdaa067. 32642717 10.1093/noajnl/vdaa067 PMC7332240

[22] Bao ZS , Chen HM, Yang MY, et al. RNA-seq of 272 gliomas revealed a novel, recurrent PTPRZ1-MET fusion transcript in secondary glioblastomas. Genome Res. 2014;24(11):1765–1773. 25135958 10.1101/gr.165126.113 PMC4216918

[23] Hu H , Mu Q, Bao Z, et al. Mutational landscape of secondary glioblastoma guides MET-targeted trial in brain tumor. Cell. 2018;175(6):1665–1678 e1618. 30343896 10.1016/j.cell.2018.09.038

[24] Navis AC , van Lith SA, van Duijnhoven SM, et al. Identification of a novel MET mutation in high-grade glioma resulting in an auto-active intracellular protein. Acta Neuropathol. 2015;130(1):131–144. 25862637 10.1007/s00401-015-1420-5 PMC4469304

[25] Singh D , Chan JM, Zoppoli P, et al. Transforming fusions of FGFR and TACC genes in human glioblastoma. Science. 2012;337(6099):1231–1235. 22837387 10.1126/science.1220834 PMC3677224

[26] Frattini V , Pagnotta SM, Tala, et al. A metabolic function of FGFR3-TACC3 gene fusions in cancer. Nature. 2018;553(7687):222–227. 29323298 10.1038/nature25171 PMC5771419

[27] Di Stefano AL , Fucci A, Frattini V, et al. Detection, characterization, and inhibition of FGFR-TACC fusions in IDH wild-type glioma. Clin Cancer Res. 2015;21(14):3307–3317. 25609060 10.1158/1078-0432.CCR-14-2199 PMC4506218

[28] Sepúlveda-Sánchez JM , Vaz MÁ, Balañá C, et al. Phase II trial of dacomitinib, a pan-human EGFR tyrosine kinase inhibitor, in recurrent glioblastoma patients with EGFR amplification. Neuro Oncol. 2017;19(11):1522–1531. 28575464 10.1093/neuonc/nox105 PMC5737732

[29] Lassman A , Sepúlveda-Sánchez J, Timothy T, et al. Infigratinib (BGJ398) in patients with recurrent gliomas with fibroblast growth factor receptor (FGFR) alterations: a multicenter phase II study. Neuro Oncol. 2019;21(Supplement_6):vi20-vi20.

[30] Cleary JM , Reardon DA, Azad N, et al. A phase 1 study of ABT-806 in subjects with advanced solid tumors. Invest New Drugs. 2015;33(3):671–678. 25895099 10.1007/s10637-015-0234-6

[31] Du XJ , Li XM, Cai LB, et al. Efficacy and safety of nimotuzumab in addition to radiotherapy and temozolomide for cerebral glioblastoma: a phase II multicenter clinical trial. J Cancer. 2019;10(14):3214–3223. 31289592 10.7150/jca.30123 PMC6603389

[32] Cloughesy T , Finocchiaro G, Belda-Iniesta C, et al. Randomized, double-blind, placebo-controlled, multicenter phase II study of onartuzumab plus bevacizumab versus placebo plus bevacizumab in patients with recurrent glioblastoma: efficacy, safety, and hepatocyte growth factor and O6-methylguanine-DNA methyltransferase biomarker analyses. J Clin Oncol. 2017;35(3):343–351. 27918718 10.1200/JCO.2015.64.7685

[33] Lemmon MA , Schlessinger J. Cell signaling by receptor tyrosine kinases. Cell. 2010;141(7):1117–1134. 20602996 10.1016/j.cell.2010.06.011 PMC2914105

[34] Snuderl M , Fazlollahi L, Le LP, et al. Mosaic amplification of multiple receptor tyrosine kinase genes in glioblastoma. Cancer Cell. 2011;20(6):810–817. 22137795 10.1016/j.ccr.2011.11.005

[35] Szerlip NJ , Pedraza A, Chakravarty D, et al. Intratumoral heterogeneity of receptor tyrosine kinases EGFR and PDGFRA amplification in glioblastoma defines subpopulations with distinct growth factor response. Proc Natl Acad Sci USA. 2012;109(8):3041–3046. 22323597 10.1073/pnas.1114033109 PMC3286976

[36] Kong DS , Song SY, Kim DH, et al. Prognostic significance of c-Met expression in glioblastomas. Cancer. 2009;115(1):140–148. 18973197 10.1002/cncr.23972

[37] Lasorella A , Sanson M, Iavarone A. FGFR-TACC gene fusions in human glioma. Neuro Oncol. 2017;19(4):475–483. 27852792 10.1093/neuonc/now240 PMC5464372

[38] Craig CG , Tropepe V, Morshead CM, Reynolds BA, Weiss S, van der Kooy D. In vivo growth factor expansion of endogenous subependymal neural precursor cell populations in the adult mouse brain. J Neurosci. 1996;16(8):2649–2658. 8786441 10.1523/JNEUROSCI.16-08-02649.1996 PMC6578757

[39] Doetsch F , Petreanu L, Caille I, Garcia-Verdugo JM, Alvarez-Buylla A. EGF converts transit-amplifying neurogenic precursors in the adult brain into multipotent stem cells. Neuron. 2002;36(6):1021–1034. 12495619 10.1016/s0896-6273(02)01133-9

[40] Wiesner SM , Decker SA, Larson JD, et al. De novo induction of genetically engineered brain tumors in mice using plasmid DNA. Cancer Res. 2009;69(2):431–439. 19147555 10.1158/0008-5472.CAN-08-1800 PMC2701484

[41] Camacho CV , Todorova PK, Hardebeck MC, et al. DNA double-strand breaks cooperate with loss of Ink4 and Arf tumor suppressors to generate glioblastomas with frequent Met amplification. Oncogene. 2015;34(8):1064–1072. 24632607 10.1038/onc.2014.29 PMC4167163

[42] Cheng SY , Huang HJ, Nagane M, et al. Suppression of glioblastoma angiogenicity and tumorigenicity by inhibition of endogenous expression of vascular endothelial growth factor. Proc Natl Acad Sci USA. 1996;93(16):8502–8507. 8710899 10.1073/pnas.93.16.8502 PMC38701

[43] Stark-Vance V . Bevacizumab and CPT-11 in the treatment of relapsed malignant glioma. Neuro Oncol. 2005;7(342):369.

[44] Gilbert MR , Dignam JJ, Armstrong TS, et al. A randomized trial of bevacizumab for newly diagnosed glioblastoma. N Engl J Med. 2014;370(8):699–708. 24552317 10.1056/NEJMoa1308573 PMC4201043

[45] Wedge SR , Kendrew J, Hennequin LF, et al. AZD2171: a highly potent, orally bioavailable, vascular endothelial growth factor receptor-2 tyrosine kinase inhibitor for the treatment of cancer. Cancer Res. 2005;65(10):4389–4400. 15899831 10.1158/0008-5472.CAN-04-4409

[46] Batchelor TT , Duda DG, di Tomaso E, et al. Phase II study of cediranib, an oral pan-vascular endothelial growth factor receptor tyrosine kinase inhibitor, in patients with recurrent glioblastoma. J Clin Oncol. 2010;28(17):2817–2823. 20458050 10.1200/JCO.2009.26.3988 PMC2903316

[47] Hao Z , Sadek I. Sunitinib: the antiangiogenic effects and beyond. Onco Targets Ther. 2016;9:5495–5505. 27660467 10.2147/OTT.S112242 PMC5021055

[48] de Boüard S , Herlin P, Christensen JG, et al. Antiangiogenic and anti-invasive effects of sunitinib on experimental human glioblastoma. Neuro Oncol. 2007;9(4):412–423. 17622648 10.1215/15228517-2007-024 PMC1994098

[49] Kreisl TN , Smith P, Sul J, et al. Continuous daily sunitinib for recurrent glioblastoma. J Neurooncol. 2013;111(1):41–48. 23086433 10.1007/s11060-012-0988-z

[50] Tamura R , Tanaka T, Akasaki Y, Murayama Y, Yoshida K, Sasaki H. The role of vascular endothelial growth factor in the hypoxic and immunosuppressive tumor microenvironment: perspectives for therapeutic implications. Med Oncol. 2020;37(1):2. 10.1007/s12032-019-1329-2 31713115

[51] van den Bent MJ , Brandes AA, Rampling R, et al. Randomized phase II trial of erlotinib versus temozolomide or carmustine in recurrent glioblastoma: EORTC brain tumor group study 26034. J Clin Oncol. 2009;27(8):1268–1274. 19204207 10.1200/JCO.2008.17.5984 PMC2667826

[52] Thiessen B , Stewart C, Tsao M, et al. A phase I/II trial of GW572016 (lapatinib) in recurrent glioblastoma multiforme: clinical outcomes, pharmacokinetics and molecular correlation. Cancer Chemother Pharmacol. 2010;65(2):353–361. 19499221 10.1007/s00280-009-1041-6

[53] Uhm JH , Ballman KV, Wu W, et al. Phase II evaluation of gefitinib in patients with newly diagnosed Grade 4 astrocytoma: Mayo/North Central Cancer Treatment Group Study N0074. Int J Radiat Oncol Biol Phys. 2011;80(2):347–353. 20510539 10.1016/j.ijrobp.2010.01.070 PMC5753591

[54] Barf T , Kaptein A. Irreversible protein kinase inhibitors: balancing the benefits and risks. J Med Chem. 2012;55(14):6243–6262. 22621397 10.1021/jm3003203

[55] Engelman JA , Zejnullahu K, Gale CM, et al. PF00299804, an irreversible pan-ERBB inhibitor, is effective in lung cancer models with EGFR and ERBB2 mutations that are resistant to gefitinib. Cancer Res. 2007;67(24):11924–11932. 18089823 10.1158/0008-5472.CAN-07-1885

[56] Zahonero C , Aguilera P, Ramírez-Castillejo C, et al. Preclinical test of dacomitinib, an irreversible EGFR inhibitor, confirms its effectiveness for glioblastoma. Mol Cancer Ther. 2015;14(7):1548–1558. 25939761 10.1158/1535-7163.MCT-14-0736

[57] Li D , Ambrogio L, Shimamura T, et al. BIBW2992, an irreversible EGFR/HER2 inhibitor highly effective in preclinical lung cancer models. Oncogene. 2008;27(34):4702–4711. 18408761 10.1038/onc.2008.109 PMC2748240

[58] Solca F , Dahl G, Zoephel A, et al. Target binding properties and cellular activity of afatinib (BIBW 2992), an irreversible ErbB family blocker. J Pharmacol Exp Ther. 2012;343(2):342–350. 22888144 10.1124/jpet.112.197756

[59] Lee JC , Vivanco I, Beroukhim R, et al. Epidermal growth factor receptor activation in glioblastoma through novel missense mutations in the extracellular domain. PLoS Med. 2006;3(12):e485. 17177598 10.1371/journal.pmed.0030485 PMC1702556

[60] Reardon DA , Nabors LB, Mason WP, et al. Phase I/randomized phase II study of afatinib, an irreversible ErbB family blocker, with or without protracted temozolomide in adults with recurrent glioblastoma. Neuro Oncol. 2015;17(3):430–439. 25140039 10.1093/neuonc/nou160 PMC4483093

[61] Cross DA , Ashton SE, Ghiorghiu S, et al. AZD9291, an irreversible EGFR TKI, overcomes T790M-mediated resistance to EGFR inhibitors in lung cancer. Cancer Discov. 2014;4(9):1046–1061. 24893891 10.1158/2159-8290.CD-14-0337 PMC4315625

[62] Kwatra M , Nanni C, Roberts C, Kwatra S, Gilbert MR, Lesser GJ. EXTH-46. A precision medicine approach to target EGFRvIII in GBM: osimertinib (AZD9291) inhibits the growth of egfrviii-positive glioblastoma stem cells and increases survival of mice bearing intracranial EGFRvIII-positive GBM. Neuro Oncol. 2017;19(Suppl 6):vi82.

[63] Li S , Schmitz KR, Jeffrey PD, Wiltzius JJ, Kussie P, Ferguson KM. Structural basis for inhibition of the epidermal growth factor receptor by cetuximab. Cancer Cell. 2005;7(4):301–311. 15837620 10.1016/j.ccr.2005.03.003

[64] Goldstein NI , Prewett M, Zuklys K, Rockwell P, Mendelsohn J. Biological efficacy of a chimeric antibody to the epidermal growth factor receptor in a human tumor xenograft model. Clin Cancer Res. 1995;1(11):1311–1318. 9815926

[65] Talavera A , Friemann R, Gómez-Puerta S, et al. Nimotuzumab, an antitumor antibody that targets the epidermal growth factor receptor, blocks ligand binding while permitting the active receptor conformation. Cancer Res. 2009;69(14):5851–5859. 19584289 10.1158/0008-5472.CAN-08-4518

[66] Eller JL , Longo SL, Hicklin DJ, Canute GW. Activity of anti-epidermal growth factor receptor monoclonal antibody C225 against glioblastoma multiforme. Neurosurgery. 2002;51(4):1005–13; discussion 1013. 12234411 10.1097/00006123-200210000-00028

[67] Eller JL , Longo SL, Kyle MM, Bassano D, Hicklin DJ, Canute GW. Anti-epidermal growth factor receptor monoclonal antibody cetuximab augments radiation effects in glioblastoma multiforme in vitro and in vivo. Neurosurgery. 2005;56(1):155–62; discussion 162. 15617598 10.1227/01.neu.0000145865.25689.55

[68] Combs SE , Schulz-Ertner D, Roth W, Herold-Mende C, Debus J, Weber KJ. In vitro responsiveness of glioma cell lines to multimodality treatment with radiotherapy, temozolomide, and epidermal growth factor receptor inhibition with cetuximab. Int J Radiat Oncol Biol Phys. 2007;68(3):873–882. 17544000 10.1016/j.ijrobp.2007.03.002

[69] Hasselbalch B , Lassen U, Hansen S, et al. Cetuximab, bevacizumab, and irinotecan for patients with primary glioblastoma and progression after radiation therapy and temozolomide: a phase II trial. Neuro Oncol. 2010;12(5):508–516. 20406901 10.1093/neuonc/nop063 PMC2940618

[70] Sivasubramanian A , Chao G, Pressler HM, Wittrup KD, Gray JJ. Structural model of the mAb 806-EGFR complex using computational docking followed by computational and experimental mutagenesis. Structure. 2006;14(3):401–414. 16531225 10.1016/j.str.2005.11.022

[71] Panousis C , Rayzman VM, Johns TG, et al. Engineering and characterisation of chimeric monoclonal antibody 806 (ch806) for targeted immunotherapy of tumours expressing de2-7 EGFR or amplified EGFR. Br J Cancer. 2005;92(6):1069–1077. 15770208 10.1038/sj.bjc.6602470 PMC2361945

[72] Scott AM , Lee FT, Tebbutt N, et al. A phase I clinical trial with monoclonal antibody ch806 targeting transitional state and mutant epidermal growth factor receptors. Proc Natl Acad Sci USA. 2007;104(10):4071–4076. 17360479 10.1073/pnas.0611693104 PMC1805701

[73] Fleming TP , Saxena A, Clark WC, et al. Amplification and/or overexpression of platelet-derived growth factor receptors and epidermal growth factor receptor in human glial tumors. Cancer Res. 1992;52(16):4550–4553. 1322795

[74] Druker BJ , Tamura S, Buchdunger E, et al. Effects of a selective inhibitor of the Abl tyrosine kinase on the growth of Bcr-Abl positive cells. Nat Med. 1996;2(5):561–566. 8616716 10.1038/nm0596-561

[75] O’Brien SG , Guilhot F, Larson RA, et al. Imatinib compared with interferon and low-dose cytarabine for newly diagnosed chronic-phase chronic myeloid leukemia. N Engl J Med. 2003;348(11):994–1004. 12637609 10.1056/NEJMoa022457

[76] Raymond E , Brandes AA, Dittrich C, et al. Phase II study of imatinib in patients with recurrent gliomas of various histologies: a European Organisation for Research and Treatment of Cancer Brain Tumor Group Study. J Clin Oncol. 2008;26(28):4659–4665. 18824712 10.1200/JCO.2008.16.9235 PMC2653126

[77] Dresemann G , Weller M, Rosenthal MA, et al. Imatinib in combination with hydroxyurea versus hydroxyurea alone as oral therapy in patients with progressive pretreated glioblastoma resistant to standard dose temozolomide. J Neurooncol. 2010;96(3):393–402. 19688297 10.1007/s11060-009-9976-3

[78] Song K , Yuan Y, Lin Y, et al. ERBB3, IGF1R, and TGFBR2 expression correlate with PDGFR expression in glioblastoma and participate in PDGFR inhibitor resistance of glioblastoma cells. Am J Cancer Res. 2018;8(5):792–809. 29888103 PMC5992513

[79] Sang Y , Hou Y, Cheng R, et al. Targeting PDGFRα-activated glioblastoma through specific inhibition of SHP-2-mediated signaling. Neuro Oncol. 2019;21(11):1423–1435. 31232447 10.1093/neuonc/noz107 PMC6827835

[80] Gherardi E , Birchmeier W, Birchmeier C, Vande Woude G. Targeting MET in cancer: rationale and progress. Nat Rev Cancer. 2012;12(2):89–103. 22270953 10.1038/nrc3205

[81] Cui JJ , Tran-Dubé M, Shen H, et al. Structure based drug design of crizotinib (PF-02341066), a potent and selective dual inhibitor of mesenchymal-epithelial transition factor (c-MET) kinase and anaplastic lymphoma kinase (ALK). J Med Chem. 2011;54(18):6342–6363. 21812414 10.1021/jm2007613

[82] Ou SH , Bartlett CH, Mino-Kenudson M, Cui J, Iafrate AJ. Crizotinib for the treatment of ALK-rearranged non-small cell lung cancer: a success story to usher in the second decade of molecular targeted therapy in oncology. Oncologist. 2012;17(11):1351–1375. 22989574 10.1634/theoncologist.2012-0311 PMC3500356

[83] Tasaki T , Fujita M, Okuda T, et al. MET expressed in Glioma stem cells is a potent therapeutic target for Glioblastoma Multiforme. Anticancer Res. 2016;36(7):3571–3577. 27354625

[84] Liu X , Wang Q, Yang G, et al. A novel kinase inhibitor, INCB28060, blocks c-MET-dependent signaling, neoplastic activities, and cross-talk with EGFR and HER-3. Clin Cancer Res. 2011;17(22):7127–7138. 21918175 10.1158/1078-0432.CCR-11-1157

[85] Kou J , Musich PR, Staal B, et al. Differential responses of MET activations to MET kinase inhibitor and neutralizing antibody. J Transl Med. 2018;16(1):253. 30208970 10.1186/s12967-018-1628-y PMC6134500

[86] Wen PY , Drappatz J, de Groot J, et al. Phase II study of cabozantinib in patients with progressive glioblastoma: subset analysis of patients naive to antiangiogenic therapy. Neuro Oncol. 2018;20(2):249–258. 29016998 10.1093/neuonc/nox154 PMC5777496

[87] Day EK , Sosale NG, Xiao A, Zhong Q, Purow B, Lazzara MJ. Glioblastoma cell resistance to EGFR and MET inhibition can be overcome via blockade of FGFR-SPRY2 bypass signaling. Cell Rep. 2020;30(10):3383–3396 e3387. 32160544 10.1016/j.celrep.2020.02.014 PMC7724645

[88] Guagnano V , Kauffmann A, Wöhrle S, et al. FGFR genetic alterations predict for sensitivity to NVP-BGJ398, a selective pan-FGFR inhibitor. Cancer Discov. 2012;2(12):1118–1133. 23002168 10.1158/2159-8290.CD-12-0210

[89] Gavine PR , Mooney L, Kilgour E, et al. AZD4547: an orally bioavailable, potent, and selective inhibitor of the fibroblast growth factor receptor tyrosine kinase family. Cancer Res. 2012;72(8):2045–2056. 22369928 10.1158/0008-5472.CAN-11-3034

[90] Perera TPS , Jovcheva E, Mevellec L, et al. Discovery and pharmacological characterization of JNJ-42756493 (Erdafitinib), a functionally selective small-molecule FGFR family inhibitor. Mol Cancer Ther. 2017;16(6):1010–1020. 28341788 10.1158/1535-7163.MCT-16-0589

[91] Jun HJ , Acquaviva J, Chi D, et al. Acquired MET expression confers resistance to EGFR inhibition in a mouse model of glioblastoma multiforme. Oncogene. 2012;31(25):3039–3050. 22020333 10.1038/onc.2011.474 PMC3774279

[92] Akhavan D , Pourzia AL, Nourian AA, et al. De-repression of PDGFRβ transcription promotes acquired resistance to EGFR tyrosine kinase inhibitors in glioblastoma patients. Cancer Discov. 2013;3(5):534–547. 23533263 10.1158/2159-8290.CD-12-0502 PMC3651754

[93] Meads MB , Gatenby RA, Dalton WS. Environment-mediated drug resistance: a major contributor to minimal residual disease. Nat Rev Cancer. 2009;9(9):665–674. 19693095 10.1038/nrc2714

[94] Louis DN , Perry A, Wesseling P, et al. The 2021 WHO classification of tumors of the central nervous system: a summary. Neuro Oncol. 2021;23(8):1231–1251. 34185076 10.1093/neuonc/noab106 PMC8328013

[95] Kang C , Sun Y, Zhu J, et al. Delivery of nanoparticles for treatment of brain tumor. Curr Drug Metab. 2016;17(8):745–754. 27469219 10.2174/1389200217666160728152939

